# Knocking-Down CD147/EMMPRIN Expression in CT26 Colon Carcinoma Forces the Cells into Cellular and Angiogenic Dormancy That Can Be Reversed by Interactions with Macrophages

**DOI:** 10.3390/biomedicines11030768

**Published:** 2023-03-02

**Authors:** Gabriele Feigelman, Elina Simanovich, Phillipp Brockmeyer, Michal A. Rahat

**Affiliations:** 1 Immunotherapy Laboratory, Carmel Medical Center, Haifa 3436212, Israel; 2 Ruth and Bruce Rappaport Faculty of Medicine, Technion-Israel Institute of Technology, Haifa 3109601, Israel; 3 Department of Oral and Maxillofacial Surgery, University Medical Center Göttingen, 37073 Göttingen, Germany

**Keywords:** CD147/EMMPRIN, CT26 cells, knockdown, dormancy, EMT, angiogenesis, proliferation

## Abstract

Metastasis in colorectal cancer is responsible for most of the cancer-related deaths. For metastasis to occur, tumor cells must first undergo the epithelial-to-mesenchymal transition (EMT), which is driven by the transcription factors (EMT-TFs) Snail, Slug twist1, or Zeb1, to promote their migration. In the distant organs, tumor cells may become dormant for years, until signals from their microenvironment trigger and promote their outgrowth. Here we asked whether CD147/EMMPRIN controls entry and exit from dormancy in the aggressive and proliferative (i.e., non-dormant) CT26 mouse colon carcinoma cells, in its wild-type form (CT26-WT cells). To this end, we knocked down EMMPRIN expression in CT26 cells (CT26-KD), and compared their EMT and cellular dormancy status (e.g., proliferation, pERK/pP38 ratio, vimentin expression, expression of EMT-TFs and dormancy markers), and angiogenic dormancy (e.g., VEGF and MMP-9 secretion, healing of the wounded bEND3 mouse endothelial cells), to the parental cells (CT26-WT). We show that knocking-down EMMPRIN expression reduced the pERK/pP38 ratio, enhanced the expression of vimentin, the EMT-TFs and the dormancy markers, and reduced the proliferation and angiogenic potential, cumulatively indicating that cells were pushed towards dormancy. When macrophages were co-cultured with both types of CT26 cells, the CT26-WT cells increased their angiogenic potential, but did not change their proliferation, state of EMT, or dormancy, whereas the CT26-KD cells exhibited values mostly similar to those of the co-cultured CT26-WT cells. Addition of recombinant TGFβ or EMMPRIN that simulated the presence of macrophages yielded similar results. Combinations of low concentrations of TGFβ and EMMPRIN had a minimal additive effect only in the CT26-KD cells, suggesting that they work along the same signaling pathway. We conclude that EMMPRIN is important as a gatekeeper that prevents cells from entering a dormant state, and that macrophages can promote an exit from dormancy.

## 1. Introduction

Colorectal cancer (CRC) is the third-most common type of cancer, and the second leading cause for cancer-related mortality worldwide [1]. About 20% of patients already suffer from metastatic lesions at the time of diagnosis, whereas 60% of the patients with no metastatic disease progress to develop metastasis within five years [2], with the liver and lung being the most frequent distant organs involved [1]. Surgery is the first-line treatment option, and it is provided to selected patients with respectable metastases and operable primary tumors. Inoperable tumors, residual lesions, or disease that has already spread to distant organs may require drug-based tumor therapy, frequently including VEGF inhibitors, such as bevacizumab [3]. However, in some clinical cases, treatment fails to eliminate chemotherapy-resistant quiescent tumor cells [4], and therapy (including chemotherapy, radiotherapy, targeted therapy, and/or immunotherapy) may sometimes promote escape from dormancy and the generation of metastatic lesions in remote organs [5,6]. Therefore, a better understanding of the mechanisms that regulate the entrance and exit from dormancy is needed, in the hope of identifying new opportunities for a future therapeutic approach. 

Metastatic cells can spread even in early tumor stages [7]. To allow epithelial cells to migrate to remote organs, they must first activate the epithelial-to-mesenchymal transition (EMT) program, which increases their invasive capabilities and motility. Cells that undergo EMT can detach from their neighboring cells, intravasate through the basement membrane into an adjacent blood or lymphatic vessel, extravasate, and colonize a remote organ [8]. Only a very small fraction of these circulating tumor cells (CTCs), will survive this journey. Those cells that end up lodging and colonizing the metastatic organ will become disseminating tumor cells (DTCs) that may remain dormant for months or years [9]. To overcome the quiescence-mediating signals in the remote organ [10], and to generate a metastatic lesion, the cell must re-acquire its epithelial properties through the activation of the mesenchymal-to-epithelial transition (MET) program, which enables proliferation, generation of macro-metastases, and cancer recurrence [11]. Thus, metastatic cells exhibit plasticity (epithelial–mesenchymal plasticity—EMP), and cells that co-express both epithelial and mesenchymal properties in varying degrees (known as E/M hybrids) are considered to have the greatest metastatic and aggressive potential [12,13,14]. EMT markers, such as reduced E-cadherin expression and increased vimentin expression, as well as increased EMT transcription factors (EMT-TFs), such as Snail/SNAI1, Slug/SNAI2, Zeb1, or Twist1, were shown to drive the EMT program, repress the epithelial markers, and induce a more mesenchymal phenotype associated with dormancy [15]. 

The DTCs that have successfully lodged in a distant organ, and do not immediately die by apoptosis nor continue to proliferate, receive signals from their new microenvironment that activate a dormancy program [16]. Two non-mutually-exclusive mechanisms have been proposed for tumor dormancy. Cellular dormancy results when DTCs enter the G0 phase in their cellular cycle and their proliferation is arrested. This state is accompanied by an increased resistance to drugs that preferentially target proliferating cells. Tumor mass dormancy describes a balance between cells that proliferate and cells that die by apoptosis, so that the small cluster of DTCs or micro-metastasis remains in a stable size and does not generate a detectable macro-metastatic lesion. This can be the result of a lack of sufficient blood supply (angiogenic dormancy) or pressure exerted by the immune response [12,17]. Cellular and tumor-mass dormancies are influenced by interactions with the surrounding tissue and stroma cells in the metastatic niche, through signals mediated by soluble factors or transmembranal proteins. Dormant DTCs express high levels of the orphan nuclear receptor NR2F1 that acts as a master regulator of dormancy, activating the expression of the cyclin-dependent kinase (CDK) inhibitors p27, p21 and p16, as well as other genes related to stemness (SOX2 and NANOG), quiescence, and cell survival [18,19,20]. 

Tumor-associated macrophages (TAMs) have been implicated in tumor progression and in many stages during the establishment of metastasis. TAMs can induce the EMT program by secreting cytokines, such as TGFβ1, EGF, IL-6 and TNFα [21,22], that in turn, induce the expression of the EMT-TFs. TAMs are also implicated in the seeding and growth of metastatic cells [23,24]. The abilities of TAMs to remodel the ECM by secreting matrix metalloproteinases (MMPs), to suppress other immune effector cells, and to enhance angiogenesis all contribute to the invasion, survival and colonization of metastatic cells [25,26,27]. However, the exact molecular nature of the interactions between macrophages and DTCs, which mediate the entrance or exit from dormancy, has been insufficiently studied. 

CD147/EMMPRIN is a multifunctional transmembrane glycoprotein that mediates interactions between tumor and stromal cells [28]. EMMPRIN is overexpressed in many types of tumors, and its expression is correlated with higher tumor grade and stage, occurrence of metastasis and poor prognosis [29]. EMMPRIN is first and foremost a pro-angiogenic factor, as it induces the expression of MMPs and VEGF [28,30], but its ability to interact with different proteins as a hub protein that stabilizes multiprotein complexes implicates it in the regulation of tumor cell proliferation, lactate efflux, cell adhesion, and drug resistance, as well [29,31,32,33]. EMMPRIN, which is directly regulated by Slug [34], can increase β-catenin phosphorylation, leading to enhanced Snail expression and suppressed E-cadherin membranal expression [35], suggesting that EMMPRIN is also involved in the induction of EMT. 

In this study, we hypothesized that EMMPRIN is involved in regulating the entry and exit of tumor cells from dormancy. We demonstrate that when EMMPRIN expression was knocked-down in the CT26 mouse colon carcinoma cell line (CT26-KD), the levels of the dormancy markers and the EMT-TFs were increased, whereas the proliferation and the angiogenic potential were reduced relative to the parental cells. Co-culturing these cells with RAW 264.7 macrophages reversed these effects, suggesting that EMMPRIN is involved, but is not essential, in the escape from dormancy. 

## 2. Materials and Methods

### 2.1. Cell Cultures

The murine colon carcinoma cell line CT26 (ATCC CRL-2638) was cultured in RPMI-1640 medium with 10% fetal calf serum (FCS), 1% penicillin/streptomycin, 1% non-essential amino acids, 1% amphotericin B, 1% pyruvate and 1% L-Glutamine (full medium, all reagents from Biological Industries, Beit Ha’emek, Israel). The macrophage-like RAW 264.7 cell line (ATCC TIB-71), was cultured in DMEM, with 10% FCS, 1% L-glutamine, and antibiotics. The mouse brain endothelial cell line bEND3 (ATCC CRL-2299) was cultured in high glucose DMEM, with 10% FCS, 1% penicillin/streptomycin, and 1% glutamine. 

All cells were split every 3–4 days at a ratio of 1:4 using trypsin–EDTA, and were used at passages 3–15. All cells were routinely checked for the presence of *Mycoplasma.* During the experiments, cells were seeded in serum starvation medium, to avoid the masking of signals. In some experiments, tumor cells were seeded in a 24-well plate (8 × 10^4^ cells/well) and the RAW 264.7 macrophage-like cells were co-cultured in the tumor cell serum starvation medium in inserts (0.4 μm pore size, Greiner Bio-One, Kremsmünster, Austria) at a ratio of 1:1, to prevent cell–cell contact and to allow separate extraction of RNA and protein. In experiments that established the effects of the macrophage mode of activation on tumor cells, RAW 264.7 cells (10^4^ cells) were pre-incubated for 24 h with stimulators of M1 activation (100 U/mL IFNγ and 100 ng/mL LPS), with stimulators of M2 activation (IL-4 and IL-13, 20 ng/mL each), or with no treatment. The supernatants were collected, diluted 1:1 with fresh serum-starvation medium, and added to a culture of CT26-WT or CT26-KD cells (10^4^ cells) for 48 h (Figure S1). In some experiments, the mouse-recombinant TGFβ1 (10 ng/mL, from PeproTech Asia, Rehovot, Israel), or mouse-recombinant EMMPRIN (at indicated concentrations, from R&D systems, Minneapolis, MN) were added to the single cultures and incubated for 48 h.

### 2.2. Preparation of the EMMPRIN Knocked-Down Cells

**Figure 1:**
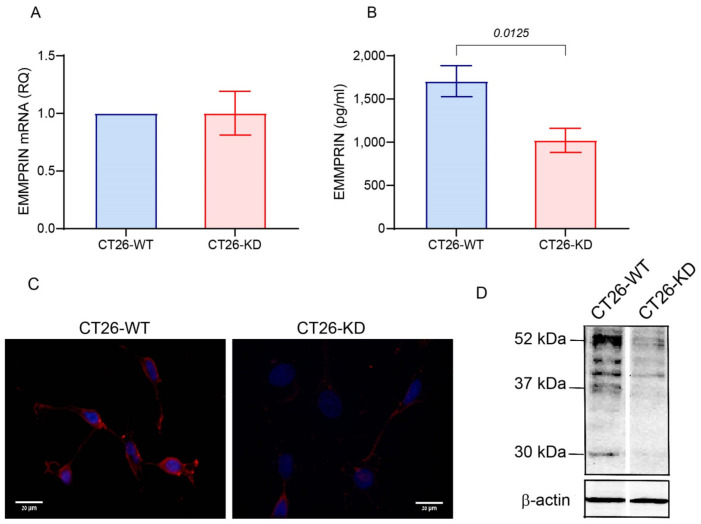
Validation of the CT26-KD cells. The parental CT26 cells (WT) and knocked-down CT26 cells (KD) were seeded (8 × 10^4^ cells each) in 24-well plates in 400 μL full medium for 48 h. At the end of the incubation, (**A**) total RNA was extracted from the cells and amplified using EMMPRIN specific primers (*n* = 4), and (**B**) the supernatants were collected for an ELISA analysis of EMMPRIN secretion (*n* = 9). Data are presented as means ± SE and analyzed using the two-tailed Student’s *t* test analysis. (**C**) CT26-WT and CT26-KD cells (30,000 cells/well/300 μL) were stained as described in the methods. A representative image, demonstrating reduced EMMPRIN protein expression in the CT26-KD cells (*n* = 3). Bar size is 20 μM. (**D**) EMMPRIN is known to appear in several bands, reflecting its low and high glycosylation patterns. Western blot analysis demonstrates that in the CT26-KD cells, all EMMPRIN bands showed a reduced expression of EMMPRIN.

To prepare CT26 cells with a reduced expression of the protein EMMPRIN, parental wildtype CT26 cells (CT26-WT) were infected with a lentivirus vector. This vector expressed four different siRNA sequences, designed specifically along the EMMPRIN sequence (Applied Biological Materials, Richmond, BC, Canada). Cells were infected at a multiplicity of infection (MOI) of 135, together with polybrene (8 µg/mL, Merck, Rahway, NJ, USA), in order to increase the efficiency of infection. Cells were incubated in a full medium, and split at a ratio of 1:3 every 4 days. As the vector also contained the puromycin resistance gene, puromycin (10 μg/mL) was added to the culture on day 6. Surviving cells that carry the lentivirus vector were grown with the puromycin selection for 3 weeks, and the medium was replaced every 3–4 days. Our vector did not contain GFP, which is immunogenic and might drive cell death [36]. To ensure that cells that express normal levels of EMMPRIN did not contaminate the CT26-KD population, a strategy of limiting dilution was applied, and cells were seeded at a concentration of one cell/well and allowed to proliferate, in order to generate sub-clones. Sub-clones were then tested for their expression of EMMPRIN at both the mRNA and protein levels (Figure 1). 

### 2.3. Cell Viability Assay

To assess viability, CT26-WT and CT26-KD cells were seeded (10^4^ cells/well) in a 96-well plate, in 100 μL of serum starvation medium, and exposed to the experimental conditions. After 48 h of incubation, the viability of the cells was assessed by adding 10 µL of the cell counting kit 8 reagent (CCK-8, Abcam, Cambridge, UK) and incubating for an additional 2 h. The water-soluble tetrazolium salt WST-8 (2-(2-methoxy-4-nitrophenyl)-3-(4-nitrophenyl)-5-(2,4-disulfophenyl)-2H-tetrazolium) was taken up and reduced in the mitochondria of viable cells to a soluble orange formazan dye, which could be measured at 450 nm with a 620 nm reference. The absorbance was directly proportional to the number of viable cells and was normalized to the experimental control.

### 2.4. Real-Time PCR

Total RNA was extracted from cultured cells using the Total RNA Purification Kit (Norgen Biotek Corp, Thorold, ON, Canada) according to the manufacturer’s instructions. RNA concentrations were determined using the NanoDrop-1000 spectrophotometer (Thermo Scientific, Waltham, MA, USA). Three micrograms of total RNA was reverse transcribed with the FIREScript RT cDNA synthesis Mix with oligo (dT) and random primer kit (Solis BioDyne, Tartu, Estonia) according to manufacturer’s instructions. A total of 80 ng of the resulting cDNA was amplified in triplicates with the 5X HOT FIREPol EvaGreen qPCR Mix Plus (Solis BioDyne) and 10 μM of the primers (Table 1), with an initial activation at 95 °C for 12 min followed by 40 cycles of 95 °C for 15 s, 60 °C for 20 s, and 72 °C for 20 s, using the StepOne system (Applied Biosystems, Foster City, CA, USA), in order to determine the mRNA expression level of the different genes or their endogenous reference genes PBGD or GAPDH. The comparative ΔΔCT method was used for relative quantification, and non-treated cells served as calibrators in each experiment, to allow for the comparison of the relative quantity (RQ) between the samples.

### 2.5. Wester Blot Analysis

Equal amounts of lysates extracted from the CT26-WT and CT26-KD cells were denatured and loaded onto a 10% SDS-polyacrylamide gel electrophoresis (15 μg/lane), and after separation, they were transferred onto pre-cut cellulose nitrate membranes (Advansta, San Jose, CA, USA). Membranes were blocked with the block-Chemi buffer (Advansta) overnight at 4 °C, and then incubated with the primary antibody (goat anti-mouse EMMPRIN, R&D systems) diluted 1:1000 for 1 h at room temperature. Afterwards the membranes were washed three times with the wash buffer (1xTBS with 0.05% Tween-20). The secondary antibody (HRP-conjugated donkey anti-goat IgG, Jackson ImmunoResearch Labs, West Grove, PA, USA) was diluted 1:5000, and incubated for 1 h at room temperature, following three washes. Then, membranes were incubated with the Western Bright ECL-HRP substrate (Advansta), and bands were visualized using the Omega Lum G Imaging System (Aplegen, Pleasanton, CA, USA). The membranes were stripped and re-probed with the mouse anti-β-actin antibody to verify equal loading. Bands were quantified using the ImageJ software. 

### 2.6. Sandwich ELISA

The mouse EMMPRIN concentrations in cell supernatants were determined using the matched antibody pair kit (Abcam, Cambridge, UK). Concentrations of secreted mouse TGFβ, TNFα, IL-6, MMP-9 and VEGF were measured with DuoSet ELISA kits (R&D Systems, Minneapolis, MN, USA). Concentrations of the phosphorylated ERK1/2 and p38α MAPKs were determined in cell lysates prepared in lysis buffer (1 mM EDTA, 0.5% Triton X-100, 5 mM NaF, 6M urea, 25 μg/mL leupeptin, 25 μg/mL pepstatin, 100 μM PMSF, 3 μg/mL aprotinin, 2.5 mM sodium pyrophosphate, 1 mM activated sodium orthovanadate in PBS, pH 7.4) using the DuoSet ELISA kits (R&D Systems). All kits were carried out according to the instructions of the manufacturers. 

### 2.7. Wound Assay

The bEND3 mouse brain micro-vessel endothelial cells were seeded in a 96-well plate (4 × 10^4^ cells/well) in 100μL of full medium and allowed to reach confluency overnight. Using a pipette tip, a scratch was applied, and the detached cells were washed away. Conditioned medium (CM), obtained from supernatants of CT26-WT or CT26-KD cells (10^5^ cells/well/300 µL) treated with the relevant cytokines or incubated in co-culture, was diluted 1:2 with full endothelial cell medium and applied to the bEND3 scratched cells. Images of the scratch at the beginning of the experiment (time 0 h) and at the end of the experiment (time 24 h) were obtained by an inverted microscope (Magnification at 4×). The length to which cells migrated to was measured using the ImagePro Plus 4.5 software (Media Cybernetics Inc., Rockville, MD, USA). 

### 2.8. Immunofluorescence (IF)

CT26-WT or CT26-KD cells (3 × 10^4^ cells) were seeded overnight on sterile cover slips in 300 μL of full media to allow their adherence. Next day, the medium was replaced with serum starvation medium supplemented with the different cytokines, as detailed in each experiment, and cells were incubated for 48 h. At the end of the experiment, cells were fixed with 300 μL of 4% formaldehyde for 10 min. Formaldehyde was washed away 3 times with PBS, and cells were then blocked (2% donkey normal serum, 0.1% Triton-X100 in PBS) for 1 hr at room temperature, and then incubated overnight at 4 °C with the following primary antibodies: rabbit anti-Vimentin (diluted 1:700, Abcam), rat anti-E-cadherin (diluted 1:200, BioLegend, San Diego, CA, USA), or goat anti-EMMPRIN (diluted 1:200, R&D systems) in 120 μL of the blocking buffer. The next day, cells were washed three times with PBS, and incubated with the corresponding secondary antibodies (diluted 1:1000): Donkey anti-Rabbit Alexa Fluor^®^ 555, Donkey anti-Rat Alexa Fluor^®^ 488 or Donkey anti-Goat Alexa Fluor^®^ 568 (all from Abcam), respectively, for 1 h in the dark at room temperature. Cells were washed once with PBS, and once with PBS containing 10 nM of DAPI for 5 min. Coverslips were mounted with FluoreGuard Mounting Medium (Hard Set) (Scytek) on carrying glass and sealed with nail polish. 

### 2.9. Statistical Analysis

All experiments were independently repeated at least four times and results are represented as mean ± standard error of mean (SEM). Statistical significance between two groups was assessed with the unpaired two-tailed Student’s *t* test, and significance between three groups or more was determined using a one-way ANOVA followed by Bonferroni’s post-hoc multiple comparison test. All tests were performed at a significance level of α = 5% using the Prism 9.5 statistical software (GraphPad, La Jolla, CA, USA).

## 3. Results

### 3.1. The Construction and Validation of the CT26 EMMPRIN-KD Cells

To learn about the involvement of EMMPRIN in the metastatic outbreak, we knocked-down its expression in the CT26 parental cell line (CT26-WT), using the lentivirus vector. After 3 weeks of continuous exposure to puromycin, we isolated cells that expressed reduced EMMPRIN levels. Since the cells did not carry the GFP marker, which is immunogenic when injected to mice and drives tumor rejection [37], we could not follow the cells or select them according to the intensity of the GFP expression. Therefore, we used a strategy of limiting dilutions, and seeded the cells at a concentration of one per well, so that the resulting clones originated from the same cells and were homogenous in their EMMPRIN expression. We then selected a clone that showed a maximal reduction in EMMPRIN expression (CT26-KD), and used it for the following experiments. 

We examined the expression of EMMPRIN at both the protein and RNA levels. We observed no change in the EMMPRIN mRNA levels compared to the CT26-WT cells (Figure 1A), whereas the protein levels within the cells (Figure 1C,D) and the secreted levels of EMMPRIN (Figure 1B) were significantly reduced. This strongly suggests that the lentivirus vector regulation on EMMPRIN expression is post-transcriptional, in agreement with our previous experiments that implicated miR-146a-5p in its regulation [38]. It is noteworthy that the levels of EMMPRIN in the CT26-KD cells are reduced by 70–86%, as assessed by the immunofluorescence and by the WB analysis, so that sufficient amounts of the protein are still present in the CT26-KD cells to allow for their viability. In the supernatants, we observed a reduction of only 40%, which could be explained by the accumulation of EMMPRIN over time. 

### 3.2. Macrophages Secrete Mediators That May Drive the Metastatic Outbreak

Macrophages have been suggested to contribute to the EMT process and to metastasis, but their contribution may strongly depend on their mode of activation [39]. TAMs have often been shown to be activated mostly as M2, and we show here, at least partially, that this holds true also for our in vitro setting. First, we show that the co-culturing of RAW 264.7 macrophages with CT26-WT tumor cells was sufficient to increase their expression of the typical M2 activation marker CD206 at the mRNA level (Figure S1A). To understand whether the macrophage mode of activation changes the effects they exert on the tumor cells, we activated RAW 264.7 macrophages for 24 h as M0 (no treatment), M1 (incubation with 100 U/mL IFNγ and 100 ng/mL LPS) or M2 (incubation with IL-4 and IL-13, 20 ng/mL each). We then collected the respective supernatants, diluted them 1:1 with fresh serum-starvation medium and added them to CT26-WT or CT26-KD cells. Relative to CT26-WT cells that were incubated with M1 supernatants, the proliferation of the same cells incubated with M0 or M2 supernatants was increased, whereas the CT26-KD cells exhibited reduced proliferation in the M0 and M2 groups and increased proliferation in the M1 group, relative to the CT26-WT cells (Figure S1B). A similar pattern was observed for the secretion of VEGF, with unchanged VEGF levels in the group receiving M1 supernatants (Figure S1C). Secretion of EMMPRIN was actually increased when CT26-WT cells were incubated with supernatants from M1-activated macrophages relative to M0- or M2-activated macrophages, in agreement with the ability of EMMPRIN to function as a pro-inflammatory protein under certain clinical settings (Figure S1D). EMMPRIN secretion was naturally reduced in all CT26-KD cells. These results suggest that the presence of tumor cells activates RAW 264.7 macrophages as M2 macrophages, and, in turn, these macrophages promote proliferation and angiogenesis in the co-cultured cells.

**Figure 2:**
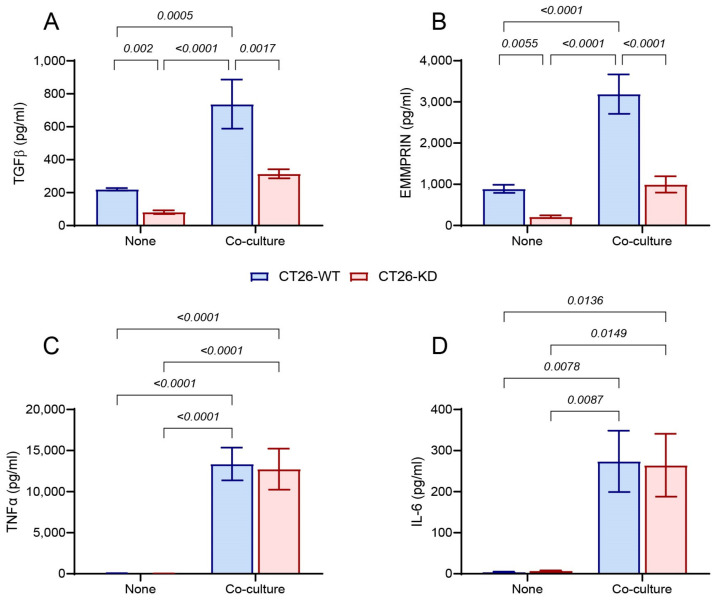
Co-culturing enhances the secretion of EMT-driver cytokines. CT26-WT or CT26-KD cells (80,000 cells each) were each cultured alone or co-cultured with RAW 264.7 cells that were seeded in the upper chamber of the inserts (0.4 μm pore size) at a ratio of 1:1, in serum-starvation medium (final volume 650 μL) for 48 h. At the end of the incubation, supernatants were collected and the concentrations of (**A**) TGFβ (*n* = 5), (**B**) soluble EMMPRIN (*n* = 6), (**C**) TNFα (*n* = 6), and (**D**) IL-6 (*n* = 6) were determined by ELISA. Data are presented as means ± SE, and analyzed using a two-way ANOVA followed by Bonferroni’s post-hoc test.

We now asked whether the co-culturing of CT26-WT and CT26-KD tumor cells with RAW 264.7 macrophages enhanced the production of cytokines associated with the metastatic outbreak. In our experimental system, the macrophages and CT26 tumor cells were separated by inserts, so that cell–cell contacts were avoided, allowing only soluble mediators to drive the change. IL-6 and TNFα are two of the inflammatory cytokines linked with the exit from dormancy [40,41,42]. We show that when both CT26-WT and CT26-KD cells were cultured alone, they produced minute amounts of these two cytokines (Figure 2C,D), but when they were co-cultured with RAW 264.7 macrophages, the secretion of both TNFα and IL-6 was markedly enhanced, suggesting that they do not depend on EMMPRIN-specific signals (Figure 2C,D). In contrast, the concentrations of both TGFβ and EMMPRIN itself in the supernatants of single cultures of CT26-KD cells were reduced compared to the single cultures of CT26-WT cells (Figure 2A,B). When CT26-KD cells were co-cultured with RAW 264.7 macrophages, these concentrations were markedly elevated compared to single cultures of CT26-KD, but were substantially lower compared to those of the CT26-WT co-cultures. While the reduced accumulation of EMMPRIN in the CT26-KD supernatants could be explained by its reduced expression in these cells, the reduced accumulation of TGFβ strongly implicates EMMPRIN in its regulation. Since we were interested in the effects of the co-culture mediated by EMMPRIN, we simulated the effect of the co-culture in the following experiments by adding either recombinant TGFβ or recombinant EMMPRIN to the tumor cells. 

### 3.3. Reduced EMMPRIN Expression Enhances Markers of Dorancy

**Figure 3:**
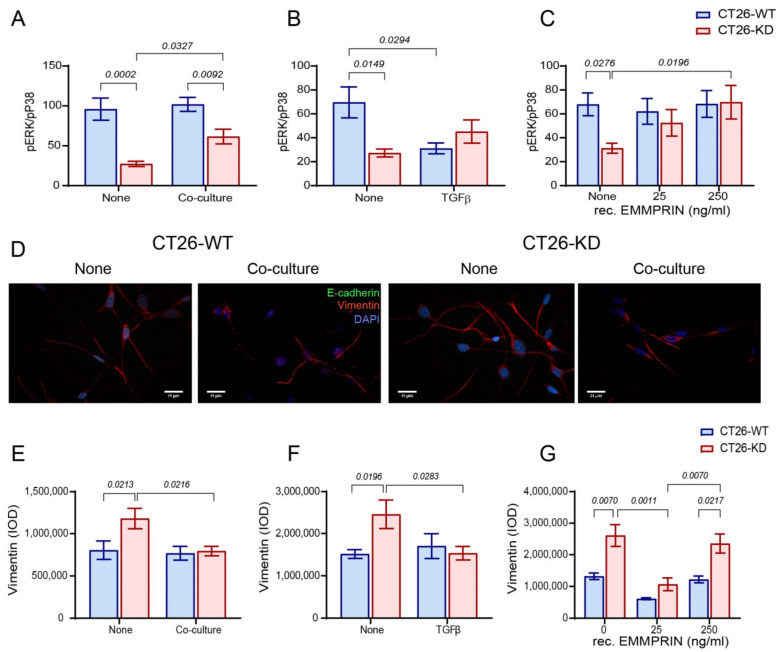
CT26-KD cells exhibit reduced levels of ERK activation and enhanced expression of vimentin, which are reversed by the co-culture or its simulation. CT26-WT or CT26-KD cells (1.5 × 10^5^ cells) were each cultured alone or in co-culture with RAW 264.7 cells that were seeded in the upper chamber of the inserts at a ratio of 1:1, in serum-starvation medium (final volume 650 μL) for 48 h. Alternatively, single cultures of CT26-WT or CT26-KD cells (1.5 × 10^5^ cells/650 μL) were cultured with or without the addition of recombinant TGFβ (10 ng/mL) or recombinant EMMPRIN (25 ng/mL or 250 ng/mL). Lysates were extracted from the CT26 cells in lysis buffer in the presence of proteinase inhibitors, and the concentrations of phosphorylated ERK1/2 and p38 were determined by ELISA. The ratio pERK/pP38 was calculated for (**A**) the co-culture (*n* = 5), (**B**) the addition of TGFβ (*n* = 5), and (**C**) the addition of recombinant EMMPRIN (*n* = 6). (**D**–**G**) CT26-WT and CT26-KD cells (3 × 10^4^ cells each) were incubated as in (**A**–**C**), mounted on cover slips, and stained for the expression of the proteins E-cadherin (green) and vimentin (red), and using DAPI (blue) to stain cell nuclei. Bar size is 25 μM. (**D**) Representative images of the staining in co-culture, quantification of vimentin expression (**E**) in the co-cultures (*n* = 6), (**F**) with addition of TGFβ (*n* = 5), and (**G**) with addition of recombinant EMMPRIN (*n* = 4). Data are presented as means ± SE, and analyzed using a two-way ANOVA followed by Bonferroni’s post-hoc test.

We hypothesized that the incubation of tumor cells in co-culture with macrophages would promote an exit from dormancy. To this end, we now measured several indicators of dormancy. CT26-WT and CT26-KD cells were incubated alone or in co-culture with RAW 264.7 macrophages. Alternatively, to simulate the effects of the co-culture and to explore as to whether they are mediated primarily by either TGFβ or EMMPRIN, we added the recombinant proteins to the single cultures of CT26-WT and CT26-KD cells. First, we assessed the ratio between the activated form of ERK1/2 and p38 MAPKs. The p38 MAPK activates a stress-like program that endows the cell with drug resistance and inhibits proliferation through the induction of the cyclin p21 and p27, whereas the ERK1/2 MAPK promotes proliferation [43]. Therefore, low ERK1/2 phosphorylation and high p38 phosphorylation indicate cellular dormancy in many cell types [43,44]. We show that, in the CT26-WT cells co-cultured with macrophages or with recombinant EMMPRIN, the pERK/pP38 ratio was unchanged relative to the single cultures, whereas the addition of TGFβ reduced it, indicating that TGFβ alone could increase p38 phosphorylation and potentially enhance dormancy, rather than exit from dormancy, in the CT26-WT cells. This was unexpected, and is only due to the increase in p38 phosphorylation, whereas ERK1/2 phosphorylation was unchanged. Since TGFβ can potentially activate both ERKs and p38 MAPK [45,46], we suggest that the assessment of the cellular dormancy state in these cells should be based on the cumulative results of all the parameters tested. In contrast, when EMMPRIN was knocked-down and its expression was reduced, CT26-KD cells exhibited a reduced pERK/pP38 ratio compared to CT26-WT, and co-culturing, or addition of TGFβ or EMMPRIN, increased this ratio, but did not return the CT26-WT levels to baseline (Figure 3A–C). Thus, the CT26-KD cells are more dormant than the CT26-WT cells, and the co-culture or its simulation (addition of TGFβ or EMMPRIN) promotes an exit from this dormant state.

Reduced E-cadherin and increased vimentin expression are the hallmarks of cells undergoing EMT and dormancy [47]. We demonstrate that neither CT26-WT nor CT6-KD cells express E-cadherin, regardless of the treatment they receive (Figure 3D and Figure S2). This is an indication that the parental CT26 cells have a more mesenchymal phenotype, as also indicated by their elongated morphology (Figure 3D) and their aggressive and proliferative behavior. Quantitation of the fluorescent signal of the vimentin expression shows that its expression was unchanged, either by the co-culture or by the addition of either TGFβ or EMMPRIN to the CT26-WT cells, (Figure 3E–G). In contrast, the CT26-KD cells cultured alone show a higher expression of vimentin, which was decreased upon co-culturing with macrophages, addition of TGFβ, or addition of a limited amount of EMMPRIN (25 ng/mL).

**Figure 4:**
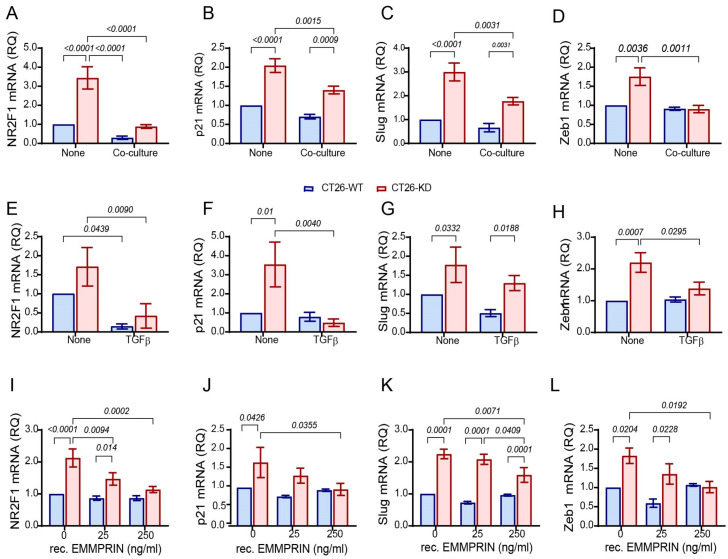
Expression of the EMT-TFs and dormancy markers is enhanced in the CT26-KD cells, but reduced in the co-culture or its simulation. CT26-WT or CT26-KD cells (8 × 10^4^ cells each) were incubated alone or in co-culture with RAW 264.7 cells as described before for 48 h. Alternatively, single cultures of CT26-WT or CT26-KD cells (8 × 10^4^ cells) were cultured with or without the addition of recombinant TGFβ (10 ng/mL) or recombinant EMMPRIN (25 ng/mL or 250 ng/mL). Total RNA was extracted from the CT26 cells, cDNA was prepared, and the genes for the dormancy markers *NR2F1* and *p21* or the EMT-TFs *Slug* and *Zeb1* were amplified by qPCR as described in the methods. (**A**–**D**) cells incubated in co-cultures (*n* = 5–6), (**E**–**H**) single cultures incubated with the addition of TGFβ (10 ng/mL) (*n* = 5–6), and (**I**–**L**) cells incubated with the addition of recombinant EMMPRIN (25 and 250 ng/mL) (*n* = 5). Data are presented as means ± SE, and analyzed using a two-way ANOVA followed by Bonferroni’s post-hoc test.

Next, we examined the gene expression of the EMT-TFs and a few markers associated with dormancy. As dormant cells must also exhibit EMT plasticity and stemness, and EMT-TFs may also drive the process of dormancy and stemness [48,49], we expected to see this reflected in the patterns of expression. Indeed, relative to the single cultures of CT26-WT cells, the expressions of the dormancy markers *NR2F1*, *p21,* and *p27* (Figure 4A,B and Figure S3B), as well as the stemness marker *SOX2* (Figure S3A) were unaffected by the co-culture, whereas the CT26-KD cells exhibited increased levels of these genes. Incubation with the macrophages reduced the expression of these genes in the CT26-KD cells, but, for the most part, their expression remained higher relative to the co-cultured CT26-WT cells. The expression of the EMT-TFs presented the same trends (Figure 4C,D and Figure S3C,D). Likewise, upon addition of TGFβ (Figure 4E–H) or EMMPRIN at higher concentrations (Figure 4I–L), the CT26-KD cells showed a reduced expression of *NR2F1*, *p21*, *Slug* and *Zeb1* genes relative to the untreated CT26-KD cells, reaching levels similar to the CT26-WT cells. These findings further support our previous conclusion that the reduced expression of EMMPRIN in the CT26-KD cells pushes the cells towards dormancy, and that the presence of macrophages can drive an exit of these cells from a dormant state. 

### 3.4. Reduced EMMPRIN Expression Inhibits Proliferation 

**Figure 5:**
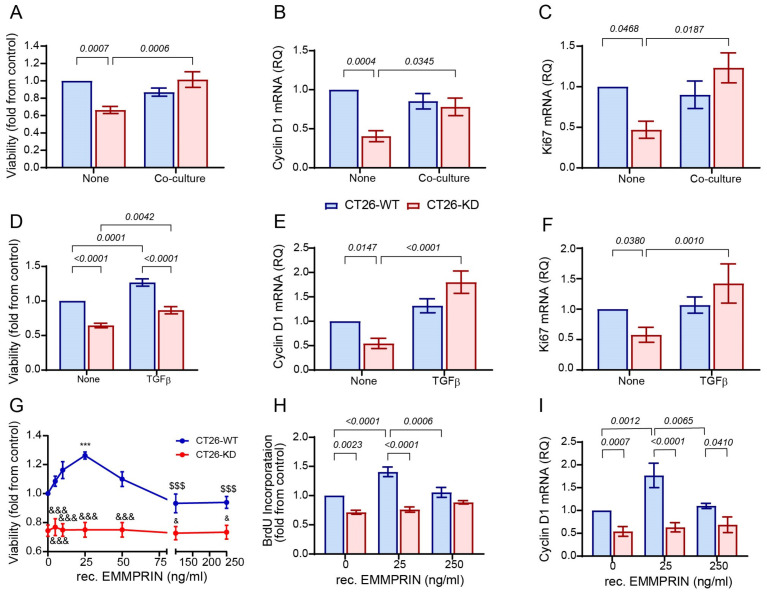
CT26-KD exhibits reduced proliferation. CT26-WT or CT26-KD cells (8 × 10^4^ cells) were incubated under the same conditions as described in Figure 4. Cell proliferation was measured using (**A**,**D**,**G**) the CCK8 kit (*n* = 8) or (**H**) the BrdU kit (*n* = 7) as well as the expression of (**B**,**E**,**I**) the cyclin D1 mRNA (*n* = 5) or (**C**,**F**), and the Ki67 mRNA (*n* = 4). (**A**–**C**) Cells incubated in co-cultures, (**D**,**F**) single cultures incubated with the addition of TGFβ (10 ng/mL), and (**G**–**I**) cells incubated with the addition of recombinant EMMPRIN (25 and 250 ng/mL). Data are presented as means ± SE, and analyzed using a two-way ANOVA followed by Bonferroni’s post-hoc test. ***, *p* < 0.001 relative to the CT26-WT without addition of rec. EMMPRIN; $$$, *p* < 0.001 relative to CT26-WT with 25 ng/ml rec. EMMPRIN; &&&, *p* < 0.001 relative to the CT26-WT at each concentration.

The most obvious manifestation of cellular dormancy is the rate of cell proliferation. To assess the proliferation of the CT26 tumor cells, we examined their viability through the CCK8 kit, that measures mitochondrial activity in viable cells, as well as the gene expression of *cyclin D1* or *Ki67*, which are associated with the cell cycle and cellular proliferation. CCK8 viability of the CT26-WT cells was not changed after co-culturing (Figure 5A), whereas the addition of TGFβ (Figure 5D) or low levels of EMMPRIN (Figure 5G) increased it. The expression of the proliferation markers *cyclin D1* and *Ki67* was not changed in the co-cultured CT26-WT cells relative to CT26-WT cells cultured alone (Figure 5B,C) or when TGFβ was added (Figure 5E,F). Interestingly, the addition of EMMPRIN enhanced the viability of the CT26-WT cells in a hormetic manner, peaking at 25 ng/mL, and then decreasing thereafter (Figure 5G), creating a bell-shaped curve. This hormetic response was also detected when incorporation of BrdU (Figure 5H) or the expression of cyclin D1 (Figure 5I) were used to detect proliferation. 

In contrast to the CT26-WT cells, the viability of the CT26-KD cells was reduced relative to the CT26-WT cells cultured alone, and co-culturing them with macrophages or adding TGFβ increased it (Figure 5A,D) to the level observed when CT26-WT cells were cultured alone. Likewise, the expression levels of cyclin D1 and Ki67 were reduced in CT6-KD cells relative to the CT26-WT controls, and co-culturing or the addition of TGFβ increased them (Figure 5B,C,E,F). However, addition of EMMPRIN at any concentration did not change the viability (Figure 5G), ability to incorporate BrdU (Figure 5H), or expression of *cyclin D1* (Figure 5I) of the CT26-KD cells. Thus, TGFβ and EMMPRIN can each enhance CT26-WT cell proliferation, whereas the co-culture probably delivers additional signals that mask their effect. In contrast, CT26-KD cells proliferate more slowly, and the macrophages or TGFβ, but not EMMPRIN, can increase the proliferation. 

### 3.5. Reduced EMMPRIN Expression Inhibits Angiogenesis

**Figure 6:**
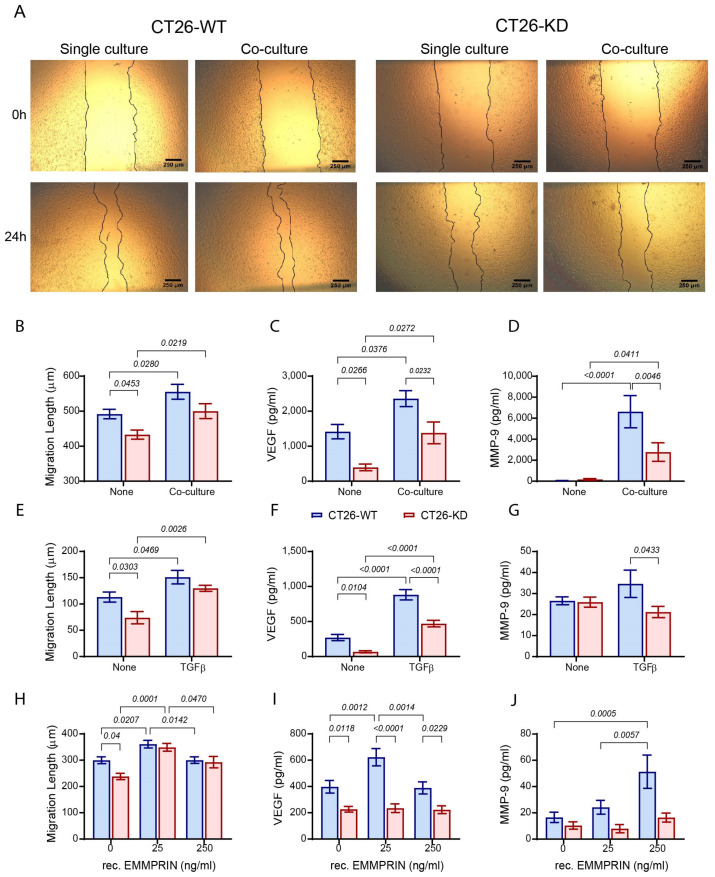
CT26-KD exhibits reduced angiogenic potential, and the co-culture reverses it. The mouse endothelial cell line bEND3 (4 × 10^4^ cells) was cultured in full medium in 96-well plates to confluency for 24 h. A scratch was made across the monolayer, detached cells were washed away, and the remaining bEND3 cells were incubated with conditioned media (CM) derived from previous experiments for 24 h, in order to allow the migration of cells to close the gap. The CM was diluted 1:2 with full medium, to a final volume of 100 μL. Images were taken before the addition of the CM (0 h) and after 24 h of incubation with the CM (24 h). (**A**) Representative images of the wound assay of co-cultures. Bar size is 250 μM. (**B**) Quantitation of the migration of bEND3 cells cultured with CM from co-culture experiments (*n* = 12), with (**C**) concentrations of VEGF (*n* = 7) and (**D**) MMP-9 (*n* = 7) in the supernatants derived from co-culture experiments. (**E**) Quantitation of the migration using CM derived from the TGFβ experiments (*n* = 5), and concentrations of (**F**) VEGF (*n* = 8) and (**G**) MMP-9 (*n* = 5) in the supernatants derived from TGFβ experiments. (**H**) Quantitation of the migration using CM derived from the recombinant EMMPRIN experiments (*n* = 9), and concentrations of (**I**) VEGF (*n* = 9) and (**J**) MMP-9 (*n* = 7) in the supernatants derived from recombinant EMMPRIN experiments. Data are presented as means ± SE, and analyzed using two-way ANOVA followed by Bonferroni’s post-hoc test.

To exit from a state of dormancy, tumor cells need to increase their metabolism, and therefore their supply of oxygen and nutrients, through angiogenesis. The angiogenic potential of the CT26 cells was assessed by the functional wound assay that demonstrated the ability of conditioned media (CM) to affect the migration of the bEND3 mouse endothelial cells, as well as by the CT26 cells’ ability to secrete the pro-angiogenic factors VEGF and MMP-9 to the CM. The CT26-WT cells enhanced their migration rate upon co-culturing, addition of TGFβ or low amounts of EMMPRIN (Figure 6B,E,H and Figure S4), the latter again exhibiting a hormetic response. A similar response was observed for the secretion of VEGF (Figure 6C,F,I). However, the secretion of MMP-9 was strongly induced in the co-culture, unchanged with the addition of TGFβ, and slightly elevated in a non-hormetic dose–response manner with the addition of EMMPRIN (Figure 6D,G,J). The strong induction of MMP-9 in the co-culture can be explained by the enhanced secretion of TNFα (Figure 2C), which is a very strong inducer of MMP-9 [50], and the relative mild response to soluble EMMPRIN at high doses demonstrates the ability of EMMPRIN to induce MMP-9 secretion from tumor cells even in the absence of TNFα.

In contrast, CM derived from the CT26-KD cells inhibited the migration of bEND3 cells, while CM derived from the co-culture, TGFβ, or EMMPRIN experiments restored migration to the levels of the similarly treated CT26-WT cells (Figure 6B,E,H). Secretion of VEGF was also reduced in the CT26-KD cells, and CM from co-cultures or from TGFβ addition, but not from the EMMPRIN experiments, increased its levels (Figure 6C,F,I), but not to the levels of the treated CT26-WT cells. As VEGF is regulated by both TGFβ and EMMPRIN, it is likely that addition of TGFβ could enhance VEGF. However, the reduced expression of the membranal EMMPRIN also reduced the homophilic interactions with the added soluble EMMPRIN, possibly leading to no effect of the latter on the CT26-KD cells. The secretion of MMP-9 from CT26-KD cells was minimal, and was increased in the CT26-KD cells only in the co-culture, but not by the addition of TGFβ or EMMPRIN. Despite the presence of equal amounts of TNFα secreted by both CT26-WT and CT26-KD in co-culture (Figure 2C), we still observed an inhibition in the MMP-9 levels in the co-cultured CT26-KD cells. This could be explained by the crosstalk with other signaling pathways, such as the ERK1/2 kinases or PI3K pathways. Thus, while the CT26-WT cells exhibit enhanced angiogenic potential when co-cultured with macrophages or simulated by TGFβ or EMMPRIN, the CT26-KD cells with reduced EMMPRIN expression demonstrated reduced angiogenic potential that was in accordance with their enhanced angiogenic dormancy. These CT26-KD cells were pushed to exit this angiogenic dormant state by stimuli provided by the co-culture, TGFβ or EMMPRIN, that were partially EMMPRIN-independent. 

### 3.6. Combined Stimulation with TGFβ and EMMPRIN Is not Sufficient to Simulate the Co-Culture

**Figure 7:**
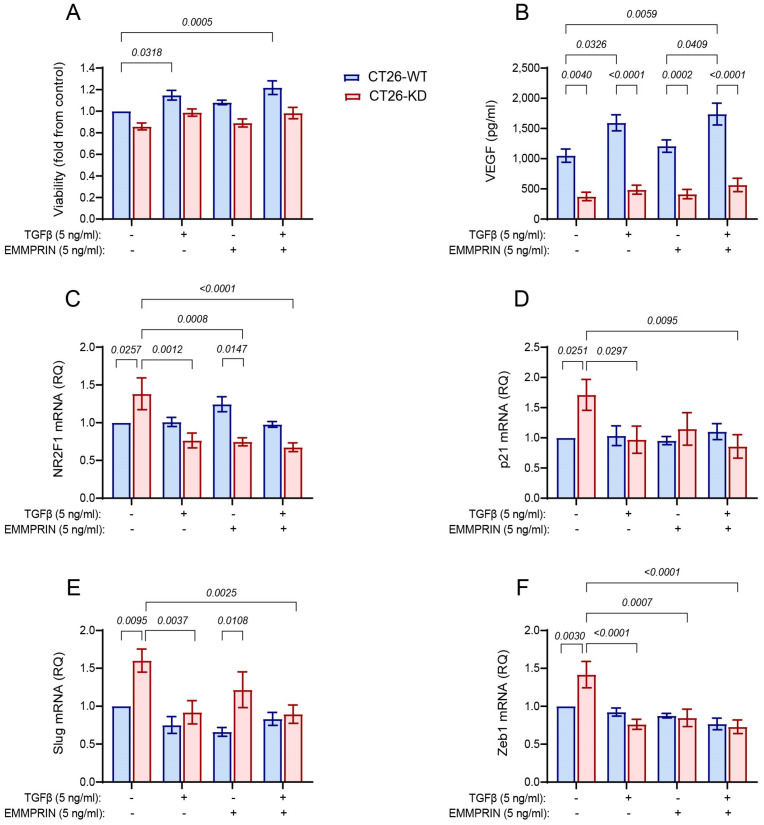
The combination of recombinant TGFβ and EMMPRIN has no effect on the addition of TGFβ or EMMPRIN alone. CT26-WT or CT26-KD cells (2.5 × 10^4^ cells) were incubated in triplicates for 48 h in serum starvation medium, with or without the addition of recombinant TGFβ (5 ng/mL), EMMPRIN (5 ng/mL), or their combination. The effect on the (**A**) proliferation, as measured by CCK8 (*n* = 10), (**B**) the concentrations of secreted VEGF (*n* = 5), as well as the mRNA expression of (**C**) *NR2F1* (*n* = 6), (**D**) *p21* (*n* = 6), (**E**) *Slug* (*n* = 6), and (**F**) *Zeb1* (*n* = 6). Data are presented as means ± SE, and analyzed using a two-way ANOVA followed by Bonferroni’s post-hoc test.

We next asked whether TGFβ and EMMPRIN could cooperate to enhance the exit from dormancy of the CT26-KD cells. To observe such possible synergism, we used sub-optimal concentrations of the two proteins and examined the effect of their combination on the different parameters used in this study. We show that while TGFβ enhanced CT26-WT’s, but not CT26-KD’s, viability, its combination with EMMPRIN had no additional effect on the viability of either CT26-WT or CT26-KD cells (Figure 7A). TGFβ, but not low levels of EMMPRIN, increased VEGF secretion from CT26-WT cells, and the combination of TGFβ and EMMPRIN resulted in a small but significant additive increase in VEGF secretion (Figure 7B). However, no change in VEGF secretion was observed in the CT26-KD cells, regardless of the stimulation. The dormancy markers *NR2F1* and *p21* and the EMT-TFs *Slug* and *Zeb1* were unchanged by the addition or combination of TGFβ and EMMPRIN in the CT26-WT cells. The expression of these genes was increased in the untreated CT26-KD cells as observed before, and the addition of TGFβ, EMMPRIN or their combination similarly reduced them to the levels of the stimulated CT26-WT cells (Figure 7C–F). Thus, the combination of TGFβ and EMMPRIN had no effect, relative to the addition of TGFβ or EMMPRIN alone, in both the CT26-WT and CT26-KD cells, suggesting that the two proteins may work along the same signaling pathway to regulate the exit from dormancy. 

## 4. Discussion

This study demonstrates the importance of EMMPRIN as a gatekeeper that prevents tumor cells from entering a dormant state. First, we show that tumor cells alone can modulate the activation of untreated macrophages, and shift them towards M2 macrophages that enhance the expression of the characteristic M2 activation maker CD206. Moreover, incubation with the tumor cells for 48 h is sufficient to induce the secretion of macrophage-derived factors that enhance tumor cell proliferation and secretion of VEGF. This suggests that the metastatic cells actively modulate their neighbouring macrophages to become M2-activated, and thereby gradually, and over time, change the immediate microenvironment to accommodate their needs. Moreover, this is, at least partially, mediated by EMMPRIN, as CT26-KD cells exhibit a reduced response. 

Data also suggests that EMMPRIN is only partially required for mediating the interactions with macrophages that are needed to exit dormancy. We moreover show that several changes occur upon knocking-down EMMPRIN expression, including inhibition in TGFβ production, reduction in the pERK/pP38 ratio, and the enhancement of vimentin expression, dormancy markers and EMT-TFs. Additionally, proliferation and the angiogenic potential of the cells are inhibited. Thus, the single intrinsic change of knocking down EMMPRIN expression is sufficient to drive cells into a dormant state, affecting both cellular and angiogenic dormancy. 

When macrophages are co-cultured with CT26-KD cells, all the above-mentioned indicators return to their parental WT values, even if this is not achieved completely. The pERK/pP38 ratio is increased, but does not reach the levels of co-cultured CT26-WT cells, the expression of vimentin, dormancy markers, and EMT-TFs is reduced almost to the levels of the co-cultured CT26-WT cells, and proliferation and angiogenic potential are increased, reaching the CT26-WT levels when cultured alone. Therefore, when EMMPRIN expression is reduced, co-culturing with macrophages can drive CT26-KD cells out of dormancy. This could be due to the residual expression of EMMPRIN in the CT26-KD cells, which may be sufficient to drive the exit from dormancy when extrinsic signals are received, or due to the influence of additional signaling pathways triggered by the co-culture, such as the pathways induced by the increased levels of IL-6 and TNFα levels, which may be at play. Note that these two options can co-exist, and are not mutually exclusive. Therefore, the ability of macrophages to promote an exit from dormancy depends only partially on the presence of EMMPRIN. 

Macrophages are producers of inflammatory cytokines in the TME, and especially of cytokines such as TGFβ, IL-6 and TNFα that have been recognized as drivers of the EMT process [51]. Moreover, inflammation has already been implicated in having a role in the exit from dormancy [52], in agreement with our findings. Here we show that the co-culture enhanced the secretion of TGFβ, IL-6 and TNFα, but only TGFβ, and not TNFα or IL-6, was reduced in the CT26-KD cells by the reduced expression of EMMPRIN. This suggests that TGFβ and EMMPRIN regulate each other. Indeed, a positive feedback loop has been described between these two proteins in hepatocellular carcinoma cells, whereby TGFβ induces EMMPRIN expression through its effects on *Slug*, and EMMPRIN can enhance TGFβ bioavailability through its impact on MMPs that cleave pro-TGFβ and also increase TGFβ transcription through β-catenin [53,54]. We suggest that such a loop also exists in CT26 colon carcinoma cells. 

The process of EMT and its reversal by MET are both necessary for successful metastatic outgrowth. In fact, the expression of the EMT-TFs (e.g., Snail, Slug, Zeb), stem-like markers (e.g., NANOG, SOX2), and dormancy markers (e.g., NR2F1, p21, p27) was shown to be linked, as dormant cells need to arrest their cell cycle and maintain the stem-like properties that allow them to survive for long periods, and then self-renew upon receiving the appropriate signals from the microenvironment [47]. We show here that the CT26-WT cells do not show any significant change in proliferation and in the expression of vimentin, EMT-TFs and dormancy markers when co-cultured with macrophages, although the angiogenic potential was increased in these co-cultured cells. In contrast, the addition of recombinant TGFβ or low levels of recombinant EMMPRIN caused an increase in proliferation and in the angiogenic potential, but not in the EMT-TFs and those dormancy markers that we examined. We suggest that these results indicate that while TGFβ and EMMPRIN are sufficient in determining the EMT and dormancy status of the cell, other pathways, perhaps the PI3K/Akt pathway [55], need to cooperate to determine the state of proliferation and angiogenesis. The lack of change in most of the EMT and dormancy markers upon the addition of TGFβ, or low levels of EMMPRIN in the CT26-WT cells, may suggest that additional factors (e.g., FGFs, IGF, ROS, S100A8/9 [56]) that are lacking when only recombinant proteins are added may be needed to enhance the production of the EMT and dormancy markers in the CT26-WT tumor cells. We did not look for such factors in our analyses, and this should be elaborated on in a following study. Alternatively, this lack of change in the co-culture vs. single culture of CT26-WT can be explained by the known aggressiveness of the CT26 cells [57,58], such that the untreated cells already express high, almost maximal basal levels of these markers. Therefore, any increase in their expression in the co-cultured cells is only marginal and not significant. However, the increase in proliferation and angiogenic potential indicate that the addition of TGFβ or low levels of EMMPRIN is sufficient to induce these pathways. 

We propose that EMMPRIN serves as a gatekeeper to prevent entry into a dormant state, and that when EMMPRIN levels are reduced in the CT26-KD cells, not only are the dormancy markers elevated, but also the EMT-TFs levels are increased. This is in contradiction to previous reports that demonstrated EMMPRIN as a protein that promotes EMT and the expression of the EMT-TFs Snail and Slug [34,35,59,60]. For example, in the LNCaP prostate cancer cell line, knockdown of EMMPRIN resulted in an increased expression of E-cadherin and decreased expression of β-catenin and Snail [35]. In the Huh7 and HepG2 HCC cells, overexpression of EMMPRIN resulted in increased expression of MMPs that cleaved and activated TGFβ, leading to the induced expression of Snail and Slug, the latter being a regulator of EMMPRIN expression, again demonstrating the positive feedback loop between TGFβ and EMMPRIN [34,53]. To explain this discrepancy, we suggest that the state of ERK/p38 activation in the CT26 cells is crucial to the final outcome. ERK1/2 activation has been shown to cooperate with the TGFβ signaling to induce the expression of EMT-TFs [45]. Mutations in the RAS gene, which is upstream of ERK1/2, and specifically the RAS^G12D^ mutation, markedly enhance Snail expression [61]. The fact that CT26 cells harbor a RAS^G12D^ mutation [58] probably contributes to the activation of ERK1/2 and its cooperation with the TGFβ pathway, so that the EMT-TFs are enhanced in the CT26-WT cells. However, when EMMPRIN expression is reduced and the positive feedback loop is inhibited, TGFβ expression is also reduced. We expected that this would lead to downregulation of EMT-TFs, but, surprisingly, we found their enhanced expression instead. We suggest that this might be due to the decreased pERK/pP38 ratio in the CT26-KD cells relative to the CT26-WT cells, which was the result of both decreased ERK phosphorylation and increased p38 phosphorylation. As EMMPRIN can activate the ERK1/2 signaling [62], we propose that in the context of reduced TGFβ signaling and decreased ERK1/2 activation, increased p38 activation can enhance the expression of the EMT-TFs, specifically of *Slug* and *Snail*, as has been shown before [63,64], as well as the dormancy markers *SOX2*, *p21* and p27 [65,66]. This hypothesis suggests that EMMPRIN has a role in integrating several signaling pathways, including TGFβ and RTKs, to determine the outcome of the metastatic cell. Whether EMMPRIN knockdown is sufficient to induce cellular dormancy, and whether p38 activation is really at the heart of this change, requires more detailed investigation using additional cell lines. 

Of interest, the addition of recombinant EMMPRIN to CT26 cells emphasizes the difference between membranal EMMPRIN that acts as a receptor, and soluble EMMPRIN that acts as a ligand. Although soluble EMMPRIN has been shown to bind to membranal EMMPRIN via homophilic interactions [29,67], it may also bind to other receptors. For proliferation, which represents cellular dormancy, and for wound healing and VEGF secretion, which represent angiogenic dormancy, recombinant EMMPRIN acts in a hormetic manner, resulting in a bell-shaped dose–response curve. These cellular functions depend on the presence of membranal EMMPRIN functioning as a receptor, because the knocked-down cells lose this hormetic pattern of behavior. The hormetic response of the CT26-WT to soluble EMMPRIN suggests that EMMPRIN signaling pathways are complex. It is unclear as to what may be the advantage in the hermetic response for the tumor cell. However, since EMMPRIN serves as a hub for many proteins and signaling pathways [32], we can speculate that this hermetic response may be a mechanism of adaptation or protection against higher levels of soluble EMMPRIN, which might be secreted into the circulation from a very large primary tumor, or during a chronic inflammatory response.

## 5. Conclusions

We show here that EMMPRIN prevents cells from entering a dormant state, and that knocking down EMMPRIN expression pushes them toward dormancy. Co-culturing of the CT26-KD cells that exhibit reduced EMMPRIN expression, with macrophages, resulted in their exit from the dormant state, although EMMPRIN filled only a partial role in this escape from dormancy. The detailed study of all the signaling pathways involved and their possible crosstalk merits a more detailed investigation, not only in the CT26 cell line, but in other types of cancerous cells as well. Additionally, our findings suggest that targeting EMMPRIN expression in cancer cells as a new therapeutic approach might push metastatic cells towards dormancy, and prevent the danger of cancer relapse.

## Figures and Tables

**Table 1:** List of primers used for qPCR amplification.

Gene Amplified	Forward Primer	Reversed Primer
*EMMPRIN*	5′-TGGCCTTCACGCTCTTGAG	5′-CAACGCCACTGCTGTTCAAA
*Snail*	5′-TGTCTGCACGACCTGTGGAAAG	5′-CTTCACATCCGAGTGGGTTTGG
*Slug*	5′-TCTGTGGCAAGGCTTTCTCCAG	5′-TGCAGATGTGCCCTCAGGTTTG
*Twist1*	5′-GATTCAGACCCTCAAACTGGCG	5′-AGACGGAGAAGGCGTAGCTGAG
*Zeb1*	5′-ATTCAGCTACTGTGAGCCCTGC	5′-CATTCTGGTCCTCCACAGTGGA
*SOX2*	5′-AACGGCAGCTACAGCATGATGC	5′-CGAGCTGGTCATGGAGTTGTAC
*NR2F1*	5′-CCAACAGGAACTGTCCCATCGA	5′-CCGTTTGTGAGTGCATACTGGC
*p21*	5′-TCGCTGTCTTGCACTCTGGTGT	5′-CCAATCTGCGCTTGGAGTGATAG
*p27*	5-AGCAGTGTCCAGGGATGAGGAA	5′-TTCTTGGGCGTCTGCTCCACAG
*Cyclin D1*	5′-GCAGAAGGAGATTGTGCCATCC	5′-AGGAAGCGGTCCAGGTAGTTCA
*c-Myc*	5′-TCGCTGCTGTCCTCCGAGTCC	5′-GGTTTGCCTCTTCTCCACAGAC
*Ki-67*	5′-GAGGAGAAACGCCAACCAAGAG	5′-TTTGTCCTCGGTGGCGTTATCC
*PBGD*	5′-CAGTTTGAAATCATTGCTATGTCCA	5′-CTCCAATCTTAGAGAGTGCAGTATC
*GAPDH*	5′- CATCACTGCCACCCAGAAGACTG	5′- ATGCCAGTGAGCTTCCCGTTCAG

## Data Availability

The data presented in this study are available in the article and Supplementary Materials.

## Supplementary Materials

The following supporting information can be downloaded at: https://www.mdpi.com/article/10.3390/biomedicines11030768/s1. Figure S1: The CT26-WT activates RAW 264.7 macrophage as M2-macrophages to promote proliferation and angiogenesis; Figure S2: CT26-KD cells exhibit enhanced expression of vimentin that is reduced by a simulation of a co-culture with RAW 264.7 cells; Figure S3: Expression of additional EMT-TFs and dormancy markers in the CT26 cells incubated in co-culture; Figure S4: The CT26-KD cells exhibited reduced angiogenic potential, and addition of recombinant TGFβ or EMMPRIN reversed it.
