# Supplementary material for: Knocking-Down CD147/EMMPRIN Expression in CT26 Colon Carcinoma Forces the Cells into Cellular and Angiogenic Dormancy That Can Be Reversed by Interactions with Macrophages
Source: Biomedicines. 2023 Mar 2;11(3):768. doi: 10.3390/biomedicines11030768 (PMC10044868; doi:10.3390/biomedicines11030768)
Supplement: Supplementary file 1 [file biomedicines-11-00768-s001.zip › biomedicines-2249042-supplementary.pdf]

Supplementary Material

1 Supplementary Figures

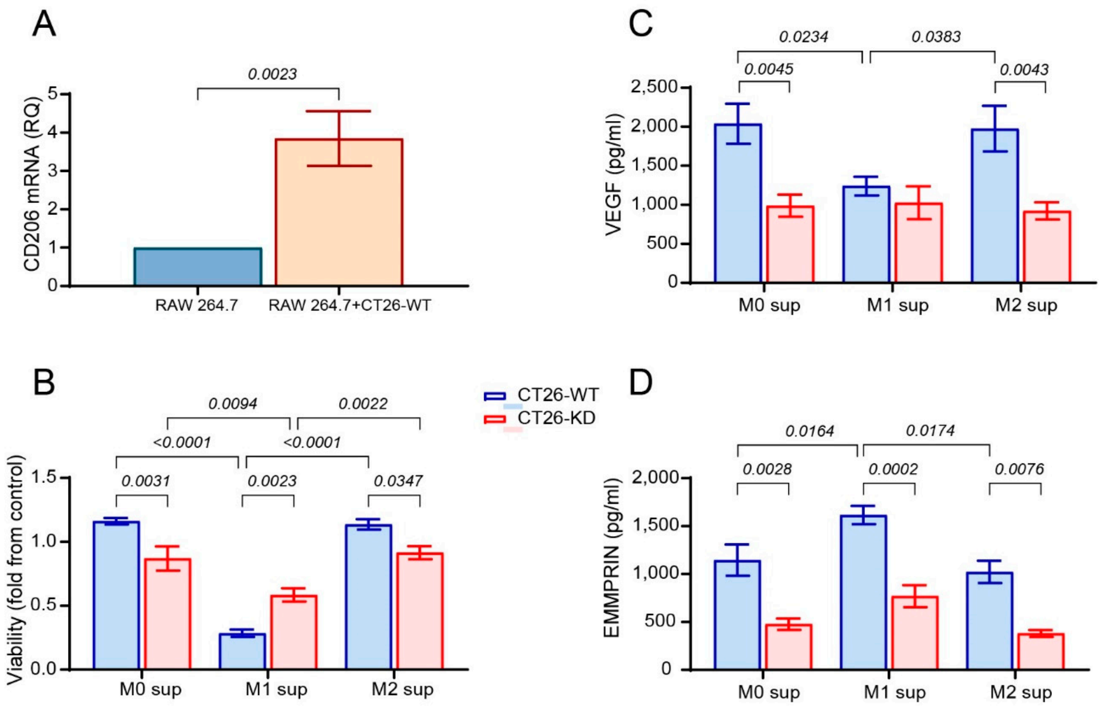

Supplementary Figure S1. The CT26-WT activates RAW 264.7 macrophage as M2-macrophages to promote proliferation and angiogenesis. **(A)** RAW 264.7 macrophages (2x10^5 cells) were incubated alone or with CT26-WT cells (4x10^5 cells) for 48 h in serum starvation medium. Total RNA was extracted and the *CD206* mRNA expression was determined by qPCR (n=3). Data is presented as mean ±SE and analyzed by the two-tailed paired student *t* test. **(B-D)** RAW 264.7 macrophages (10^4 cells) were incubated for 24 hours in serum starvation medium with no treatment, with the addition of IFNγ (100 U/ml) and LPS (100 ng/ml) to activate them as M1-macrophages, or with IL-4 and IL-13 (20 ng/ml each) to activate them as M2-macrophages. Supernatants were collected, diluted 1:1 with fresh medium, and added to a culture of CT26-WT or CT26-KD cells (10^4 cells) for a 48 hour incubation. **(B)** Proliferation was evaluated by the CCK-8 kit (n=6), **(C)** VEGF and **(D)** EMMPRIN secretion were evaluated by ELISA (n=6). Data are presented as means ± SE and were analyzed using two-way ANOVA followed by the Bonferroni's post hoc test. Macrophages that were co-cultured with CT26 cells without any treatment were activated as M2-macrophages and exerted similar effects.

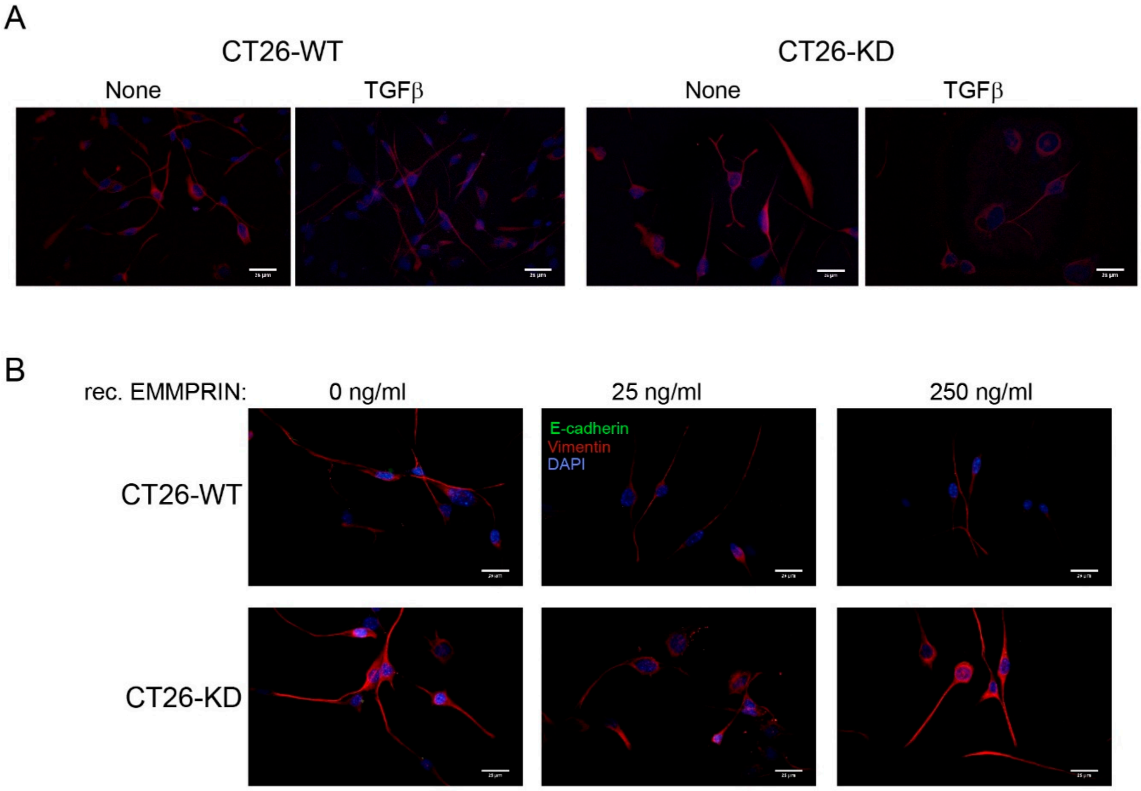

Supplementary Figure S2. CT26-KD cells exhibit enhanced expression of vimentin that is reduced by the co-culture with RAW 264.7 cells or its simulation. Single cultures of CT26-WT and CT26-KD cells ($3x10^4$ cells) were incubated on cover slips with or without the addition of recombinant TGFβ (10 ng/ml) or recombinant EMMPRIN (25 ng/ml or 250 ng/ml). Representative images of CT26-WT and CT26-KD cells that were incubated for 48h with or without the addition of recombinant **(A)** TGFβ or **(B)** EMMPRIN. At the end of the incubation, cells were stained for the expression of the proteins E-cadherin (green) and vimentin (red), and using DAPI (blue) to stain cell nuclei. Bar size is 25μM. Quantitation of these experiments is presented in figure 3.

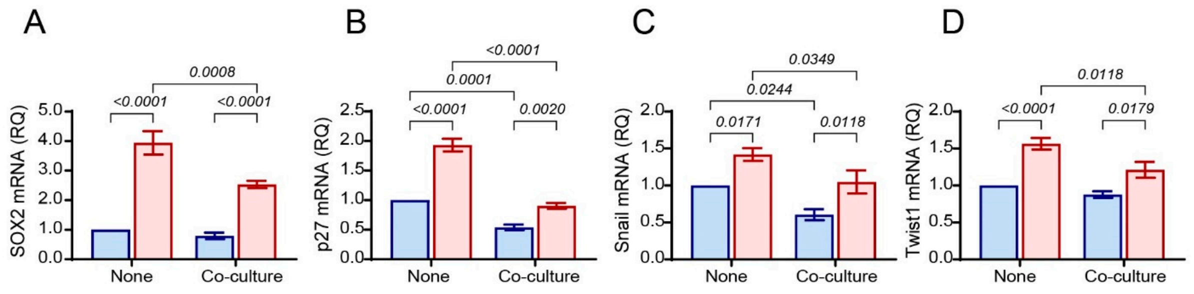

Supplementary Figure S3. Expression of additional EMT-TFs and dormancy markers in the CT26 cells incubated in co-culture. CT26-WT or CT26-KD cells ($8x10^4$ cells each) were incubated for 48 h alone or in co-culture with RAW 264.7 cells that were seeded in the upper chamber of the inserts at a ratio of 1:1, in serum-starvation medium (final volume 650µL). Total RNA was extracted from the CT26 cells, cDNA prepared, and the genes for the dormancy markers SOX2 (n=8) and p27 (n=8) or the EMT-TFs Snail (n=5) and Twist1 (n=5) were amplified by qPCR as described in the methods. Data are presented as means ± SE and analyzed using two-way ANOVA followed by Bonferroni's post-hoc test.

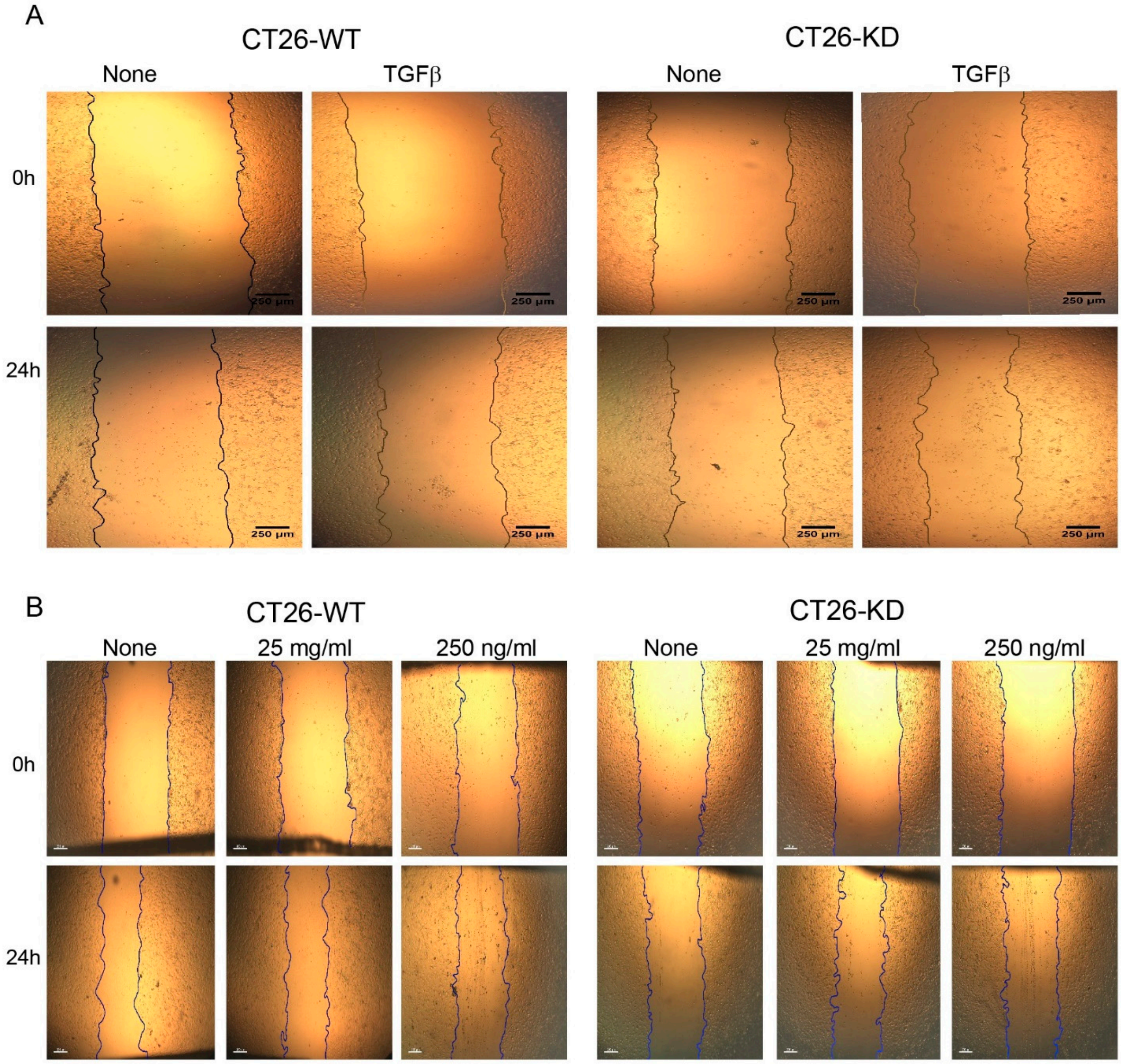

Supplementary Figure S4. **The CT26-KD cells exhibited reduced angiogenic potential, and addition of recombinant TGFβ or EMMPRIN reversed it.** The mouse endothelial cell line bEND3 ($4x10^4$ cells) were cultured in full medium in 96-well plates to confluency for 24 h. A scratch was made across the monolayer, detached cells were washed away, and the remaining bEND3 cells were incubated for 24 h with conditioned media (CM) derived from previous experiments to allow migration of the cells that closed the gap. Images were taken before the addition of the CM (0h) and after 24 h of incubation with the CM (24 h). **(A)** Representative images of the wound assay of cells cultured with CM from the addition of recombinant TGFβ experiments. Bar size is 250µM. **(B)** Representative images of the wound assay of cells cultured with CM from the addition of recombinant EMMPRIN experiments. Bar size is 200µM. Quantitation of the images is presented in figure 6.
